# Polypyridyl Zinc(II)-Indomethacin Complexes with Potent Anti-Breast Cancer Stem Cell Activity

**DOI:** 10.3390/molecules23092253

**Published:** 2018-09-04

**Authors:** Tiffany K. Rundstadler, Arvin Eskandari, Sarah M. Norman, Kogularamanan Suntharalingam

**Affiliations:** Department of Chemistry, King’s College London, London SE1 1DB, UK; tiffany.rundstadler@etu.unistra.fr (T.K.R.); arvin.eskandari@kcl.ac.uk (A.E.); sarah.norman@kcl.ac.uk (S.M.N.)

**Keywords:** metallopharmaceuticals, bioinorganic chemistry, zinc, nonsteroidal anti-inflammatory drug

## Abstract

Cancer stem cells (CSCs) are thought of as a clinically pertinent subpopulation of tumors, partly responsible for cancer relapse and metastasis. Research programs aimed at discovering anti-CSC agents have largely focused on biologics and purely organic molecules. Recently, we showed that a family of redox-active copper(II) complexes with phenanthroline-based ligands and nonsteroidal anti-inflammatory drugs (NSAIDs) such as indomethacin, are capable of potently and selectively killing breast CSCs. Herein we present analogous redox-inactive, zinc(II)-phenanthroline-indomethacin complexes with the ability to kill breast CSCs and bulk breast cancer cells with equal potency (in the submicro- or micromolar range). A single dose of the zinc(II) complexes could theoretically be administered to eliminate whole tumor populations. Excitingly, some of the zinc(II) complexes decrease the growth and viability of mammospheres to a comparable or higher degree than salinomycin, a compound known to effectively kill breast CSCs. As far as we are aware this is the first report to examine the anti-breast CSC activity of zinc(II)-containing compounds.

## 1. Introduction

Cancer is one of the most widespread illnesses in the modern world. It is a complex multigenetic condition associated with the accumulation of irregular cells, resulting from uncontrolled cell division [1]. A subpopulation of tumors, termed cancer stem cells (CSCs) is linked to metastasis and cancer relapse, the major causes of cancer associated fatalities [2,3,4]. CSCs have the ability to differentiate, self-renew, and form aggressive secondary or tertiary tumors [5,6]. Current cancer therapies (chemotherapy, radiotherapy, and surgery) are able to eliminate the bulk of cancer cells but are incapable of removing CSCs [7,8,9,10,11]. Therefore, new generations of cancer treatments must have the capability of removing entire tumor populations, including CSCs, to have durable clinical outcomes. There are many academic- and pharmaceutical-led research programs underway with the aim of developing clinically viable anti-CSC agents, however, most of them have focused on biologics or purely organic compounds [12]. We and others have recently shown that the diversity and versatility of metals can be harnessed to develop inorganic compounds with promising anti-CSC activities [13].

Platinum(II) complexes like cisplatin, carboplatin, and oxaliplatin are routinely employed in the clinic to overcome various cancers, and a lot of effort has been put into shedding light on their cellular and molecular-level mechanisms of action [14,15,16,17]. However, these platinum(II) anticancer agents are unable to remove CSCs at their therapeutically administered doses [18,19,20]. Recent efforts have shown that chemical adducts between carrier/scaffold molecules and bioactive metabolites display promising anticancer activities, albeit their anti-CSC potencies are unexplored [21,22,23,24,25]. Copper(II) complexes with phenanthroline ligands and nonsteroidal anti-inflammatory drugs (NSAIDs) such as indomethacin have been recently recognized as an effective class of anti-CSCs agents [26,27]. NSAIDs inhibit the inducible cyclooxygenase isoenzyme, COX-2, which is related to CSC renewal and proliferation [28,29,30]. The copper(II)-phenanthroline-indomethacin complexes kill breast CSCs in the micro- and sub-micromolar range, and show up to 3-fold lower potency towards bulk breast cancer cells [26,27]. Cell-based studies identified that the copper(II)-phenanthroline-indomethacin complexes prompt CSC death by increasing intracellular ROS levels (presumably via Fenton-like reactions) and inhibiting COX-2 [26,27]. The effectiveness of the copper(II)-phenanthroline-indomethacin complexes is believed to be related to the susceptibility of CSCs to variations in intracellular redox levels, and the relatively high expression of COX-2 in certain CSCs. To investigate whether redox activity is an indispensable attribute for anti-CSC activity (within this class of metal-phenanthroline-NSAID complexes), we sought to develop structurally analogous zinc(II) complexes and determine their CSC potency, given that zinc(II) is redox-inactive under physiological conditions. Herein, we present the synthesis, characterization, stability studies, and breast CSC toxicity of a family of redox-silent zinc(II)-phenanthroline-indomethacin complexes.

## 2. Results and Discussion

**Scheme 1:**
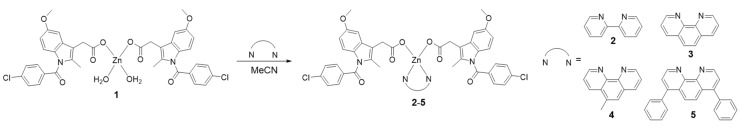
Representation of the synthetic route used to prepare the zinc(II)-phenanthroline complexes with two indomethacin units, **2**–**5**, from diaquabis(η^2^-*O*,*O*′-indomethacin)zinc(II), **1** in acetonitrile.

The zinc(II) complexes, **2**–**5** were synthesized as outlined in Scheme 1. Diaquabis(η^2^-*O*,*O*′-indomethacin)zinc(II), **1** [31] was reacted with equimolar amounts of the appropriate polypyridyl ligand (2,2′-bipyridine for **2**, 1,10-phenanthroline for **3**, 5-methyl-1,10-phenanthroline for **4**, or 4,7-diphenyl-1,10-phenanthroline for **5**) in acetonitrile. The zinc(II) complexes, **2**–**5** were obtained in good yields (70–85%) as yellow or orange solids and were fully characterized by ^1^H NMR, infrared spectroscopy, and elemental analysis (Figures S1–S6 in the Supplementary Materials). The coordination of two indomethacin moieties and one polypridyl ligand to zinc in **2**–**5** was confirmed by the 2:1, indomethacin:polypridyl ligand integration ratio in the ^1^H NMR spectra of **2**–**5** (Figures S1–S4). Furthermore, the formation of **2**–**5** was evidenced by the shift of the aromatic proton signals relative to **1** (Figure S5). According to the IR spectra of **2**–**5**, ν_asym_(CO_2_) and ν_sym_(CO_2_) stretching bands were observed at 1589–1594 cm^−1^ and 1359–1373 cm^−1^ (Figure S6). The variation in the ν_asym_(CO_2_) and ν_sym_(CO_2_) stretching bands for **2**–**5** was 221–234 cm^−1^, indicative of a monodentate binding mode for the carboxylate group on indomethacin to the zinc center (as depicted in Scheme 1) [32,33]. This is comparable to the ν_asym_(CO_2_) and ν_sym_(CO_2_) stretching band differences reported for related zinc(II) complexes containing indomethacin and polypyridyl ligands [34,35]. The high chemical purity of **2**–**5** was shown by elemental analysis studies.

The shake-flask method was used to determine the lipophilicity (Log*P*) of **2**–**5**. This involves determining the amount of a given compound partitioned between octanol and water using UV-Vis spectroscopy. The Log*P* values for **2**–**5** varied from 0.89 to 1.67 (Table S1). The size of the polypyridyl ligand has a clear effect on lipophilicity, with Log*P* values increasing according to the following order; **2** < **3** < **4** < **5**. The hydrophobicity of **2**–**5** implies that the zinc(II) complexes are likely to easily enter cells. In order to determine the stability of **2** (used as a demonstrative compound of the zinc(II) complexes) in biologically relevant solutions, UV-Vis spectroscopy studies were performed. The metal-perturbed π-π* and metal-to-ligand charge-transfer (MLCT) absorption bands of **2** (50 µM) in PBS:DMSO (200:1) were constant for 24 h at 37 °C (Figure S7), indicative of stability. The same UV-Vis bands associated to **2** (50 µM) in mammary epithelial cell growth medium (MEGM)/DMSO (200:1) remained unaltered over the course of 24 h at 37 °C, suggestive of stability in conditions required for cellular studies (Figure S8).

**Figure 1:**
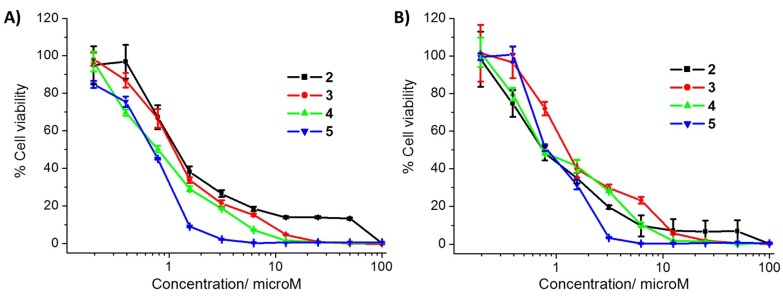
Compound concentration versus percentage cell viability plots for **2**–**5** against (**A**) bulk breast cancer cells (HMLER) and (**B**) breast CSC-like cells (HMLER-shEcad cells) (72 h incubation). The difference in potency of **2**–**5** towards HMLER-shEcad cells and HMLER cells is not significant (Student *t*-test, *p* > 0.05).

The antiproliferative effect of **2**–**5** against bulk breast cancer cells (HMLER) and breast CSC-like cells (HMLER-shEcad) was studied using the MTT assay. Dose–response curves (Figure 1A,B) were used to calculate the IC_50_ values, which are shown in Table 1. In general, the zinc(II) complexes, **2**–**5** exhibited equipotency against HMLER and HMLER-shEcad cells, in the micro- or sub-micromolar range. Therefore, **2**–**5** have the potential ability to eliminate whole tumor cell populations with a single micro- or sub-micromolar dose. Clinically approved platinum(II) agents, cisplatin and carboplatin displayed great toxicity towards CSC-depleted HMLER cells than CSC-enriched HMLER-shEcad cells, suggestive of non-CSC (bulk cancer cell) selectivity (Table 1) [27]. This is consistent with the tendency of platinum(II) anticancer agents to encourage CSC enrichment rather than CSC depletion [18]. Notably, the potency of **2**–**5** against CSC-like HMLER-shEcad cells was up to 6-fold greater than salinomycin, an anti-breast CSC compound. Previous work has shown that indomethacin is non-toxic towards bulk cancer cells and CSC-like cells (Table 1) [26]. The current results indicate that the potency of indomethacin against breast CSC-like cells and bulk breast cancer cells is improved by chelating it to a zinc(II)-polypyridyl core. The CSC potency of **2**–**5** is similar to the most effective copper(II)-phenanthroline-indomethacin complexes previously identified by us [26,27]. This shows that CSC-potent metal-phenanthroline-NSAID complexes can be developed with redox-inactive metals such as zinc(II), and implies that redox activity is not essential for anti-CSC activity within this class of compounds.

To measure therapeutic potential, the potency of **2**–**5** towards human embryonic kidney (HEK 293T) cells was investigated. The potency of **2**–**5** towards HEK 293T cells is highly dependent on the polypyridyl ligand and increases in the following order; **2** < **3** < **4** < **5** (Table 1 and Figure S9). Promisingly the 2,2′-bipyridine- and 1,10-phenanthroline-bearing complexes, **2** and **3** were significantly less potent toward HEK 293T cells (*p* < 0.05, up to 17-fold for **2**) than HMLER and HMLER-shEcad cells, demonstrating preferred toxicity towards breast cancer cells (bulk and CSC-like cells) over healthy cells.

**Figure 2:**
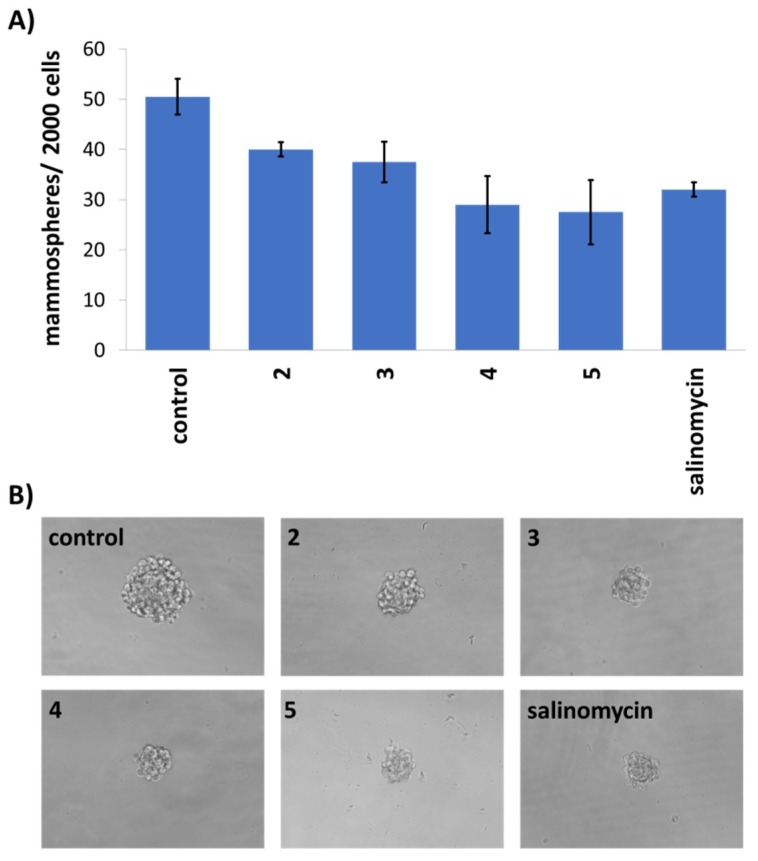

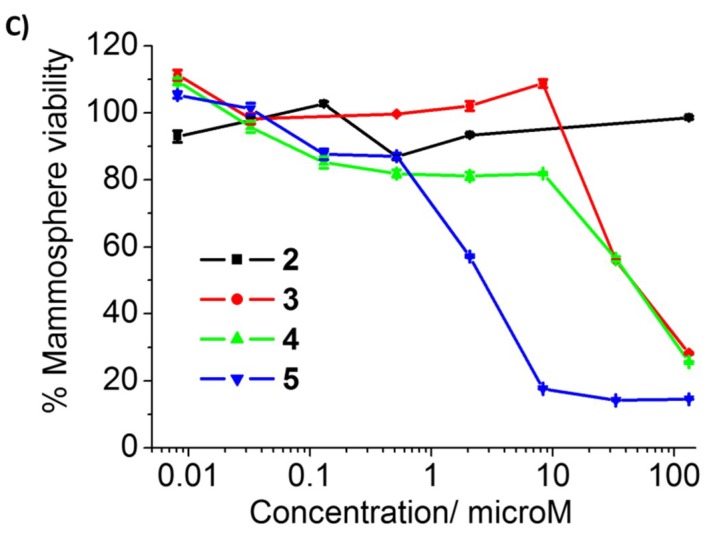
(**A**) Representation of the number of mammospheres formed from HMLER-shEcad cell suspensions treated with **2**–**5** or salinomycin for 5 days at their respective IC_20_ values. Standard deviation was used to calculate the associated errors. (**B**) Bright-field images (taken using an inverted microscope) representative of untreated HMLER-shEcad mammospheres and those treated with **2**–**5** or salinomycin for 5 days at their respective IC_20_ values (×20 magnification). (**C**) Compound concentration versus percentage mammosphere viability plots for **2**–**5** against HMLER-shEcad mammospheres (5 days incubation).

To further probe the anti-CSC potential of the zinc(II) complexes, **2**–**5**, the mammosphere assay was performed. This assay assesses the aptitude of chemical agents to stop three-dimensional spheroid formation from breast CSCs in suspension [36]. The method also provides a reliable gauge of in vivo potential, as spheroids are closer in structure to solid tumors than monolayer systems. Treatment of HMLER-shEcad cells in suspension, with **2**–**5** (at their respective IC_20_ value for 5 days) noticeably decreased the number and size of mammospheres formed (Figure 2A,B). Treatment with salinomycin under identical conditions induced a similar effect (Figure 2A,B). Notably, **4** and **5** reduced mammosphere formation (43–46% decrease in mammospheres formed) to a better degree than salinomycin (37% decrease in mammospheres formed). Previous work has shown that indomethacin has a poor mammosphere inhibitory effect [14]. TOX8, a resazurin-based compound was used to determine mammosphere viability after treatment with **2**–**5**. The zinc(II) complexes, **3**–**5** displayed micromolar potency towards mammospheres, with the 4,7-diphenyl-1,10-phenanthroline-bearing complex, **5** exhibiting 7-fold higher potency than salinomycin under identical conditions (Table 1 and Figure 2C) [37]. The ineffectiveness of **2** against mammospheres (IC_50_ > 133 µM) (Figure 2C) was surprising considering its high potency (sub-micromolar) against monolayer cell cultures of HMLER-shEcad cells (Table 1). The zinc(II) complexes, **3**–**5** displayed similar mammosphere potency to analogous copper(II)-phenanthroline-indomethacin complexes [13], indicating that redox activity may not be vital for anti-CSC activity within this class of metal-phenanthroline-NSAID complexes.

In summary we present a series of zinc(II)-phenanthroline-indomethacin complexes capable of killing bulk breast cancer cells and breast CSCs indiscriminately in the submicro- and micromolar range. Three of the zinc(II) complexes, **3**–**5** also inhibit mammosphere formation and reduce mammosphere viability to a similar or greater extent than salinomycin. Overall, the CSC potency of the redox-inactive zinc(II)-phenanthroline-indomethacin complexes is similar to the most effective redox-active copper(II)-phenanthroline-indomethacin complexes previously identified by us. This suggests that redox activity is not an essential criterion for anti-CSC activity within this class of metal-phenanthroline-NSAID complexes. Future studies will aim to elucidate the cellular mechanism of action of the zinc(II)-phenanthroline-indomethacin complexes, **2**–**5** in CSCs and determine their translatable scope.

## 3. Materials and Methods

### 3.1. General Procedures

No special conditions (i.e., nonatmospheric conditions) were used to carry out the synthetic methods reported in this manuscript. A BrukerAvance 400 MHz Ultrashield NMR spectrometer (Bruker, Billerica, MA, USA) was used to measure the ^1^H NMR spectra of **1**–**5**. An infrared (IR) Affinity-1S Shimadzu spectrophotometer (Shimadzu Corporation, Kyoto, Japan) was used to measure the IR spectra of **2**–**5**. Commercial services at London Metropolitan University were used to carry out the elemental analysis studies. 2,2′-bipyridine, 1,10-phenanthroline, 5-methyl-1,10-phenanthroline, 4,7-diphenyl-1,10-phenanthroline, and indomethacin were bought from Sigma Aldrich (St. Louis, MO, USA). Diaquabis(η^2^-*O*,*O*′-indomethacin)zinc(II), **1** was synthesized following a reported method [31]. For all biophysical and cellular studies, DMSO was used to prepare a 10 mM solution of **2**–**5**. The solutions were then diluted with the appropriate biological solution to the working concentration(s).

### 3.2. Synthesis of Zn(indomethacin)_2_(2,2′-bipyridine) (***2***)

To a solution suspension of **1** (500.3 mg, 0.61 mmol) in acetonitrile (25 mL), 2,2′-bipyridine (96.3 mg, 0.61 mmol) was added and the resulting mixture was stirred at 60 °C for 16 h. The mixture was then cooled to room temperature and the solvent was removed to ca. 10 mL. The concentrated solution was cooled to 4 °C to yield a precipitate, which as filtered and washed with water (20 mL) and ethanol (20 mL), to yield **2** as a yellow solid (440.6 mg, 77%); ^1^H NMR (400 MHz, DMSO-d_6_) δ 8.65 (d, 2H), 8.51 (d, 2H), 8.14 (t, 2H), 7.64–7.55 (m, 10H), 6.94–6.90 (m, 4H), 6.65 (dd, 2H), 3.66 (s, 6H), 3.45 (s, 4H), 2.10 (s, 6H); IR (solid state, cm^−1^): 1677, 1654, 1639, 1594, 1560, 1522, 1478, 1459, 1433, 1395, 1373, 1358, 1324, 1290, 1256, 1219, 1181, 1148, 1091, 1069, 1035, 1012, 930, 844, 836, 753, 686, 630, 551; Anal. Calcd. For C_48_H_38_Cl_2_N_4_O_8_Zn·2H_2_O: C, 59.36; H, 4.36; N, 5.77. Found: C, 59.50; H, 3.25; N, 6.06.

### 3.3 Synthesis of Zn(indomethacin)_2_(1,10-phenanthroline) (***3***)

To a solution suspension of **1** (500.3 mg, 0.61 mmol) in acetonitrile (40 mL), 1,10-phenanthroline (115.4 mg, 0.64 mmol) was added and the resulting mixture was stirred at 60 °C for 16 h. The mixture was then cooled to room temperature and the solvent was removed to ca. 10 mL. The concentrated solution was cooled to 4 °C to yield a precipitate, which as filtered and washed with water (20 mL) and ethanol (20 mL), to yield **3** as a yellow solid (423.9 mg, 72%); ^1^H NMR (400 MHz, DMSO-d_6_) δ 8.91 (bs, 2H), 8.75 (d, 2H), 8.13 (s, 2H), 7.88 (dd, 2H), 7.60 (s, 8H), 6.87–6.85 (m, 4H), 6.60 (dd, 2H), 3.59 (s, 6H), 3.40 (s, 4H), 2.05 (s, 6H); IR (solid state, cm^−1^): 1672, 1623, 1593, 1559, 1525, 1480, 1457, 1431, 1404, 1371, 1359, 1325, 1292, 1258, 1231, 1220, 1182, 1148, 1145, 1092, 1077, 1039, 1013, 926, 855, 847, 787, 757, 727, 693, 670, 629, 550; Anal. Calcd. For C_50_H_38_Cl_2_N_4_O_8_Zn: C, 62.61; H, 3.99; N, 5.84. Found: C, 62.47; H, 3.84; N, 5.96.

### 3.4. Synthesis of Zn(indomethacin)_2_(5-methyl-1,10-phenanthroline) (***4***)

To a solution suspension of **1** (500.3 mg, 0.61 mmol) in acetonitrile (25 mL), 5-methyl-1,10-phenanthroline (153.6 mg, 0.79 mmol) was added and the resulting mixture was stirred at 60 °C for 16 h. The mixture was then cooled to room temperature and the solvent was removed to ca. 10 mL. The concentrated solution was cooled to 4 °C to yield a precipitate, which as filtered and washed with water (20 mL) and ethanol (20 mL), to yield **4** as a yellow solid (418.2 mg, 70%); ^1^H NMR (400 MHz, DMSO-d_6_) δ 8.91–8.80 (m, 3H), 8.66 (d, 1H), 7.98 (s, 1H), 7.93–7.85 (m, 2H), 7.61 (s, 8H), 6.88–6.82 (m, 4H), 6.61 (dd, 2H), 3.58 (s, 6H), 3.39 (s, 4H), 2.78 (s, 3H), 2.05 (s, 6H); IR (solid state, cm^−1^): 1675, 1634, 1589, 1563, 1522, 1477, 1455, 1432, 1395, 1369, 1357, 1320, 1290, 1256, 1223, 1178, 1148, 1092, 1069, 1032, 1017, 994, 923, 837, 800, 759, 729, 669, 553; Anal. Calcd. For C_51_H_40_Cl_2_N_4_O_8_Zn·H_2_O: C, 61.80; H, 4.27; N, 5.65. Found: C, 61.43; H, 4.39; N, 5.76.

### 3.5. Synthesis of Zn(indomethacin)_2_(4,7-diphenyl-1,10-phenanthroline) (***5***)

To a solution suspension of **1** (500.3 mg, 0.61 mmol) in acetonitrile (25 mL), 4,7-diphenyl-1,10-phenanthroline (205.9 mg, 0.62 mmol) was added and the resulting mixture was stirred at 60 °C for 16 h. The mixture was then cooled to room temperature and the solvent was removed to ca. 10 mL. The concentrated solution was cooled to 4 °C to yield a precipitate, which as filtered and washed with water (20 mL) and ethanol (20 mL), to yield **5** as a orange solid (579.9 mg, 85%); ^1^H NMR (400 MHz, DMSO-d_6_) δ 8.98 (bs, 2H), 8.05 (bs, 2H), 7.87 (bs, 2H), 7.63–7.58 (m, 18H), 6.94 (bs, 2H), 6.89 (d, 2H), 6.63 (d, 2H), 3.61 (s, 6H), 3.53 (bs, 4H), 2.13 (s, 6H); IR (solid state, cm^−1^): 1683, 1671, 1593, 1562, 1523, 1476, 1456, 1433, 1398, 1359, 1320, 1289, 1261, 1222, 1183, 1144, 1093, 1070, 1039, 1015, 996, 929, 843, 832, 757, 707, 668, 632, 550; Anal. Calcd. For C_62_H_46_Cl_2_N_4_O_8_Zn: C, 67.01; H, 4.17; N, 5.04. Found: C, 66.98; H, 4.17; N, 5.16.

### 3.6. Determination of the LogP Values

The shake-flask method in combination with UV-Vis spectroscopy was used to determine the Log*P* values of **2**–**5**. Briefly, to a suspension of water (500 μL) and octanol (500 μL), **2**–**5** was added, giving a final concentration of 100 µM. The mixture was shaken for 24 h (room temperature). Using centrifugation, the water and octanol layers were separated and the concentration of **2**–**5** in each layer was determined using UV-Vis spectroscopy.

### 3.7. Cell Lines and Cell Culture Conditions

HEK 293T human embryonic kidney cells purchased from American Type Culture Collection (ATCC, Manassas, VA, USA) were grown in DMEM (Dulbecco’s Modified Eagle’s Medium) with FBS (fetal bovine serum, 10%), and penicillin/streptomycin (1%). Human mammary epithelial CSC-depleted HMLER and human mammary epithelial CSC-enriched HMLER-shEcad cells were obtained from Prof. R.A. Weinberg (Whitehead Institute, MIT). These cells were grown in MEGM (Mammary Epithelial Cell Growth Medium) with BPE, hydrocortisone, hEGF, insulin, and gentamicin/amphotericin-B. All of the cells used in this study were cultured at 37 °C within a humidified incubator with a CO_2_ controller to maintin CO_2_ levels at 5%.

### 3.8. MTT (3-(4,5-Dimethylthiazol-2-yl)-2,5-diphenyltetrazolium bromide) Assay

On day 1, 5000 cells (of the appropriate cell line) were plated in each well of a 96-well plate. The cells were then left to attach to the bottom of the wells overnight. On day 2, the compounds of interest were added (at different concentrations, 0.2–100 μM). The cells were incubated with the compounds for 72 h. The volume of each well at this stage was 200 μL. If DMSO was used to aid solubilization of the compounds, then the same amount of DMSO was also present in control wells. On day 5, a solution of 3-(4,5-dimethylthiazol-2-yl)-2,5-diphenyltetrazolium bromide (MTT) (4 mg/mL) predissolved in PBS was added to each well of the 96-well plate (20 μL). After a further 4 h incubation, the media/MTT solution was removed and the resultant purple formazan crystals were dissolved in DMSO (200 μL). The absorption of each well was measured using a plate reader at 550 nm. The absorption values were normalised according to the control wells and dose—response curves were plotted to determine the IC_50_ values. Three independent experiments were carried out, with each concentration having six repeats (n = 18).

### 3.9. Mammosphere Assay

On day 1, 5000 HMLER-shEcad cells were seeded into each well of a 96-well ultralow-attachment plate. The culture media used was MEGM with B27 (Invitrogen), 20 ng/mL EGF, and 4 μg/mL heparin (Sigma). Compounds of interest were added to the HMLER-shEcad cells at various concentrations (0–133 μM), and the suspensions were incubated for 5 days. On day 6, the mammospheres were imaged and counted using a standard microscope. Afterwhich, TOX8 (Sigma) (20 µL) was added to each well and incubated for 16 h. On day 7, the solutions in each well were transferred to a black 96-well plate and the fluorescence was measured using a plate reader, 590 nm (*λ*_ex_ = 560 nm). The arbitrary fluorescence values were normalised with respect to control wells (containing DMSO), and dose–response curves were plotted to determine the IC_50_ values. Three independent experiments were carried out, with each concentration having three repeats (n = 6).

## Figures and Tables

**Table 1:** IC_50_ values of **2**–**5**, salinomycin, cisplatin, and carboplatin against HMLER, HMLER-shEcad, and human embryonic kidney (HEK) 293T cells, and HMLER-shEcad mammospheres. Incubation times of 72 h or 120 h were used. IC_50_ values and errors represent the mean value of three independent experiments and the associated standard deviations, respectively.

Compound	HMLER IC_50_ (μM)	HMLER-shEcad IC_50_ (μM)	HEK 293T IC_50_ (μM)	Mammosphere IC_50_ (μM)
**2**	1.2 ± 0.02	0.7 ± 0.04	20.0 ± 3.9	>133
**3**	1.1 ± 0.05	1.1 ± 0.08	2.3 ± 0.1	44.1 ± 0.6
**4**	0.8 ± 0.03	0.7 ± 0.07	0.9 ± 0.2	44.2 ± 1.4
**5**	0.7 ± 0.03	0.9 ± 0.08	0.3 ± 0.02	2.7 ± 0.03
cisplatin ^1^	3.4 ± 0.5	4.9 ± 0.4	n.d.	n.d.
carboplatin ^1^	67.3 ± 2.8	72.3 ± 7.9	n.d.	n.d.
salinomycin ^1^	11.4 ± 0.4	4.2 ± 0.3	n.d.	18.5 ± 1.5

^1^ Taken from reference [26,27,37]. n.d. not determined.

## Supplementary Materials

The following are available online, Figures S1–S9.
