# Supplementary material for: Polypyridyl Zinc(II)-Indomethacin Complexes with Potent Anti-Breast Cancer Stem Cell Activity
Source: Molecules. 2018 Sep 4;23(9):2253. doi: 10.3390/molecules23092253 (PMC6225474; doi:10.3390/molecules23092253)
Supplement: Supplementary file 1 [file molecules-23-02253-s001.pdf]

Supplementary Materials for

Polypyridyl Zinc(II)-Indomethacin Complexes with Potent Antibreast Cancer Stem Cell Activity

Tiffany K. Rundstadler, Arvin Eskandari, Sarah M. Norman, and Kogularamanan Suntharalingam *

Department of Chemistry, King's College London, London, SE1 1DB, United Kingdom; tiffany.rundstadler@etu.unistra.fr (T.K.R.); arvin.eskandari@kcl.ac.uk (A.E.); sarah.norman@kcl.ac.uk (S.M.N.)

* Correspondence: kogularamanan.suntharalingam@kcl.ac.uk; Tel.: +44-207-848-2595

Received: 17 August 2018; Accepted: 31 August 2018; Published: date

Table of Content

Figure S1. ^{1}H NMR spectrum of **2** in DMSO-d_6.
Figure S2. ^{1}H NMR spectrum of **3** in DMSO-d_6.
Figure S3. ^{1}H NMR spectrum of **4** in DMSO-d_6.
Figure S4. ^{1}H NMR spectrum of **5** in DMSO-d_6.
Figure S5. ^{1}H NMR spectrum of **1** in DMSO-d_6.
Figure S6. IR spectrum of (**A**) **2**, (**B**) **3**, (**C**) **4**, and (**D**) **5** in the solid form.
Table S1. Experimentally determined Log*P* values for **2**–**5**.
Figure S7. UV-Vis spectrum of **2** (50 μM) in PBS:DMSO (200:1) over the course of 24 h at 37 °C.
Figure S8. UV-Vis spectrum of **2** (50 μM) in mammary epithelial cell growth medium (MEGM): DMSO (200:1) over the course of 72 h at 37 °C.
Figure S9. Representative dose response curves of **2**–**5** against HEK 293T cells after 72 h incubation.

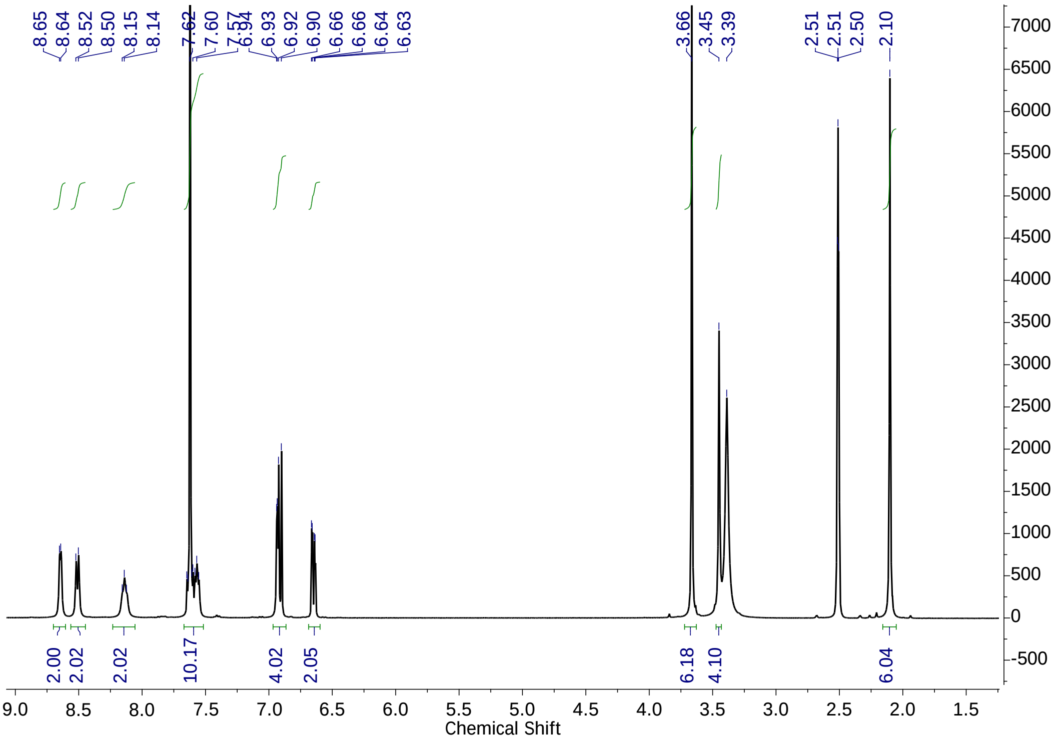

Figure S1. ^{1}H NMR spectrum of **2** in DMSO-d$_6$.

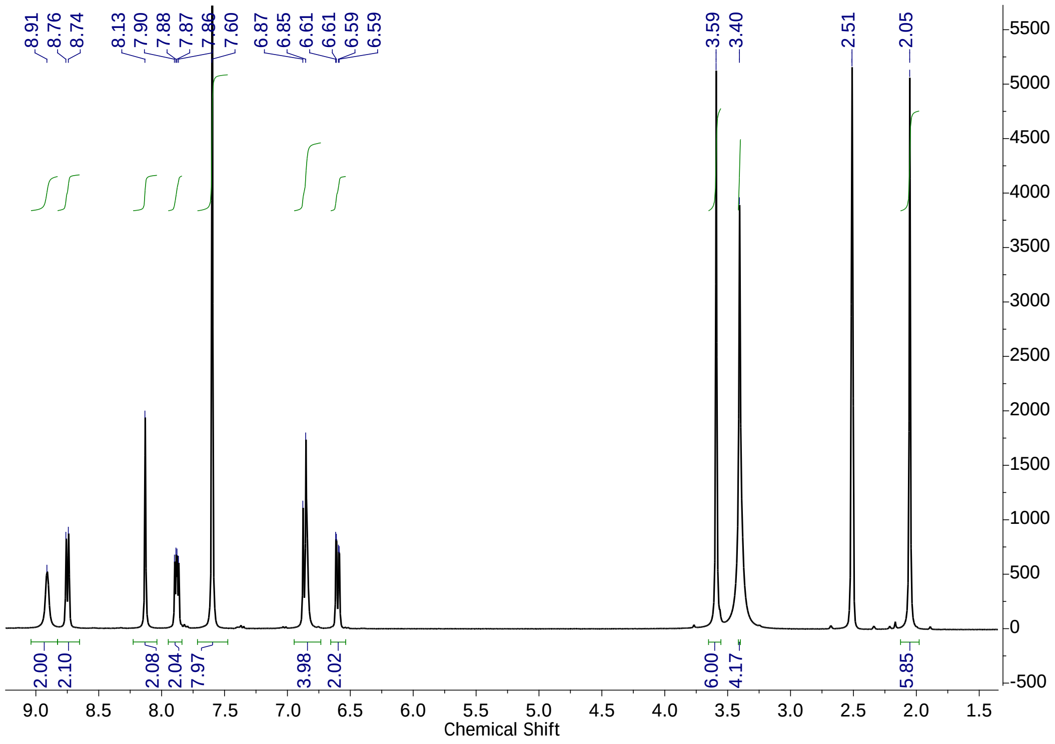

Figure S2. ^{1}H NMR spectrum of **3** in DMSO-d$_6$.

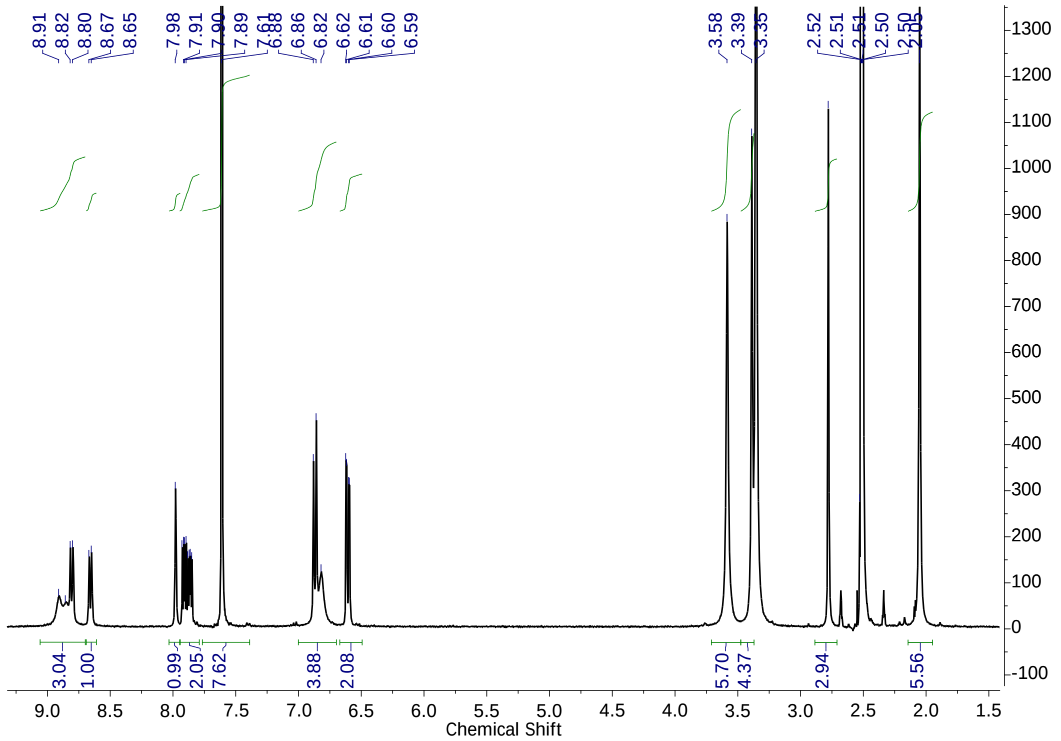

Figure S3. ^{1}H NMR spectrum of **4** in DMSO-d6.

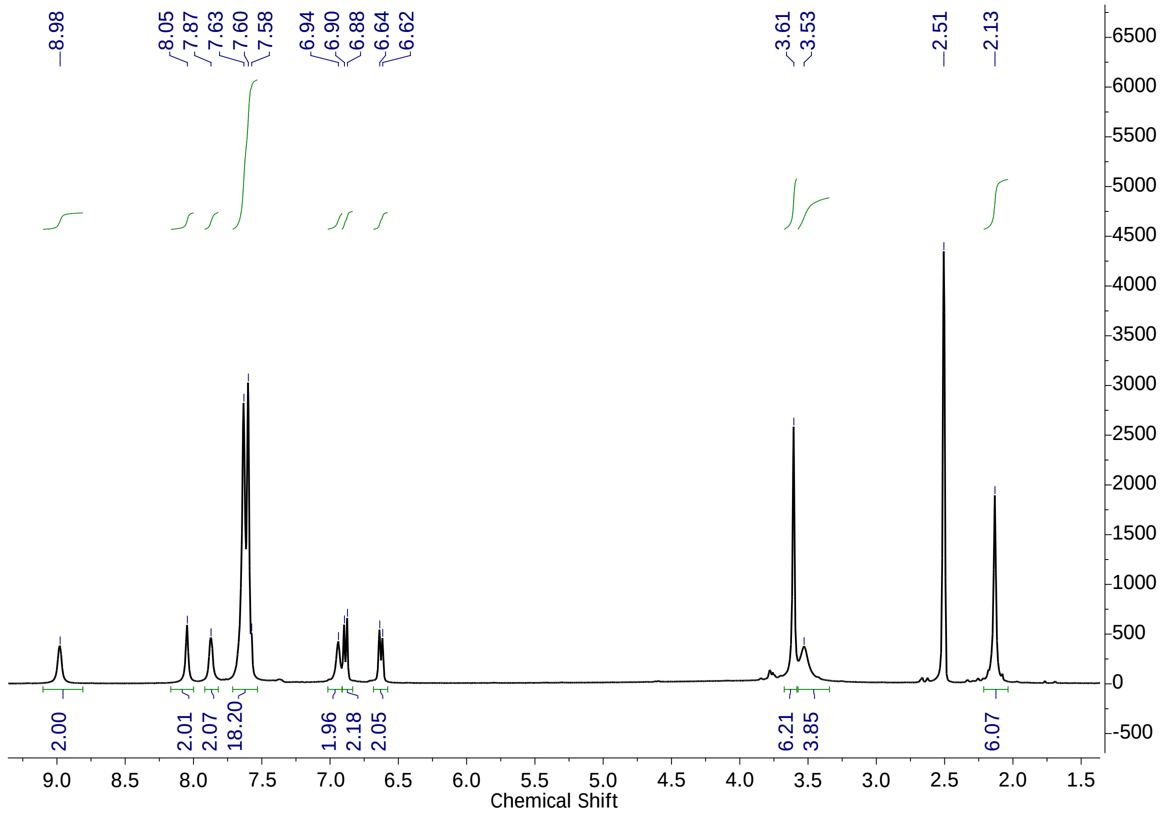

Figure S4. ^{1}H NMR spectrum of **5** in DMSO-d6.

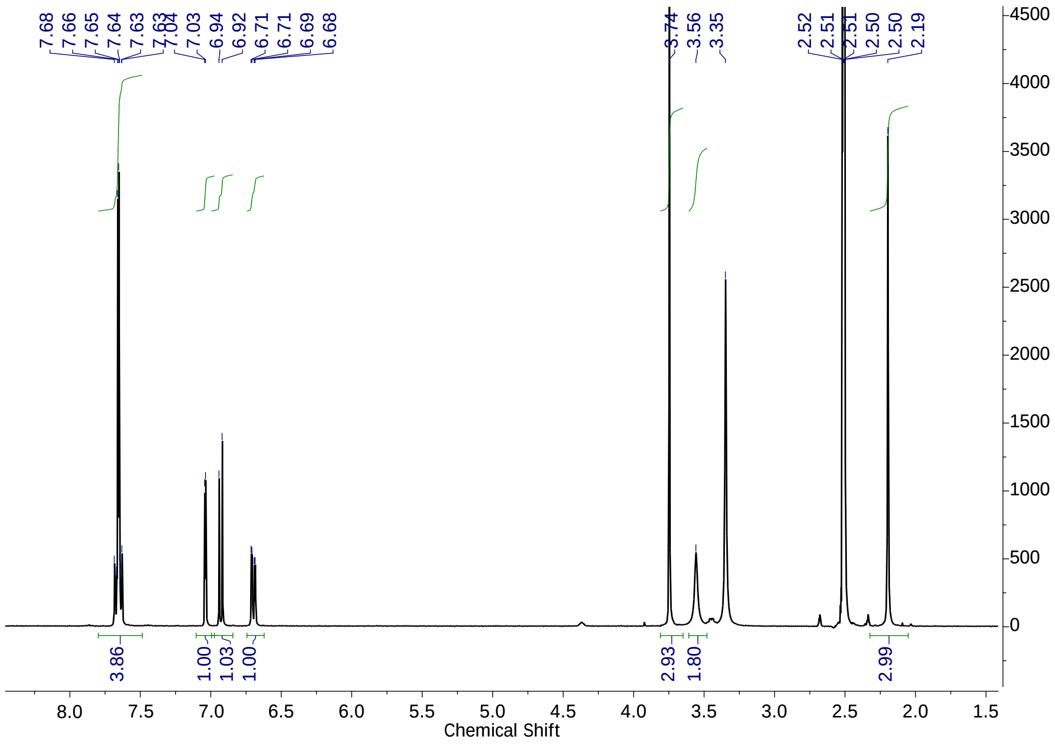

Figure S5. ^{1}H NMR spectrum of **1** in DMSO-d_6.

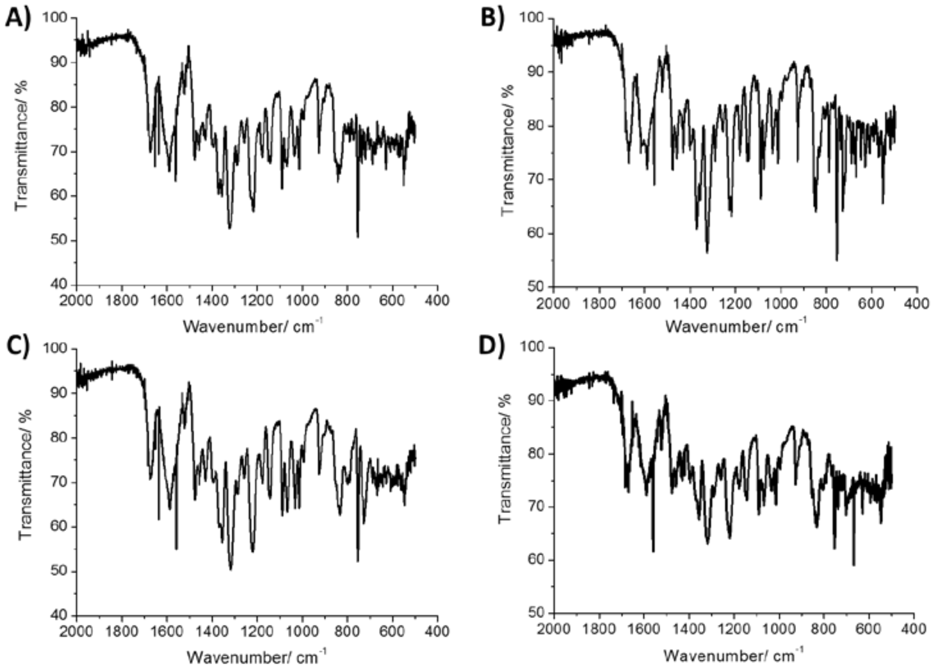

Figure S6. IR spectrum of (**A**) **2**, (**B**) **3**, (**C**) **4**, and (**D**) **5** in the solid form.

Table S1. Experimentally determined Log*P* values for **2**–**5**.

Zn(II) Complex	Log*P*
**2**	0.89
**3**	0.97
**4**	1.24
**5**	1.67

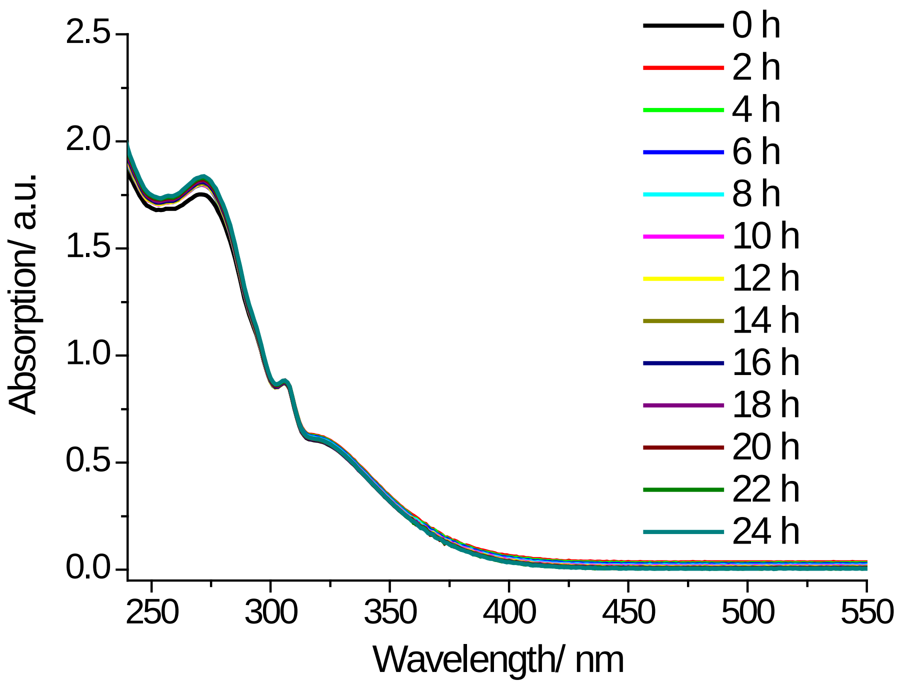

Figure S7. UV-Vis spectrum of 2 (50 μM) in PBS:DMSO (200:1) over the course of 24 h at 37 °C.

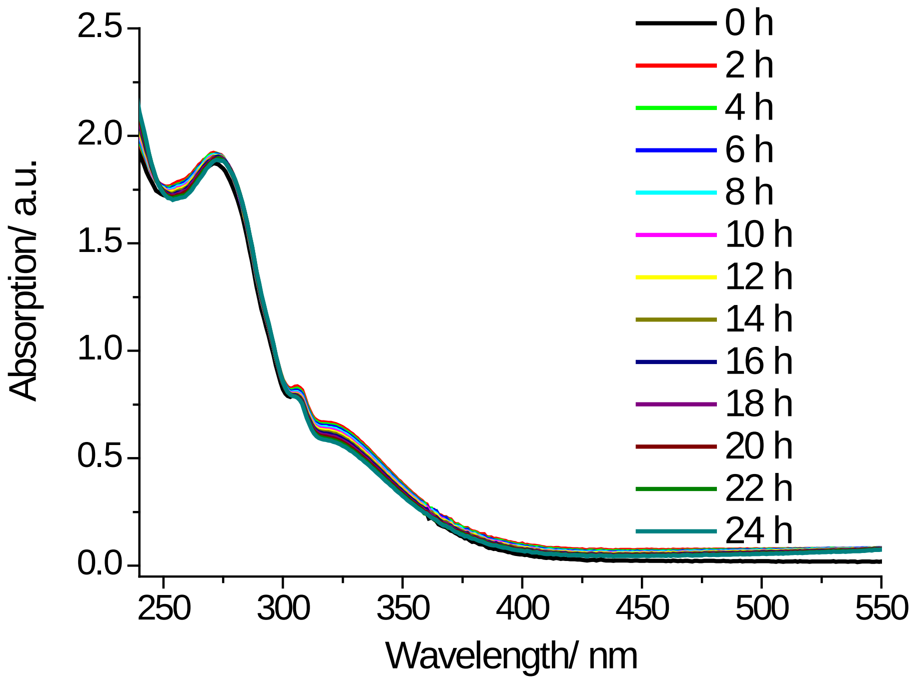

Figure S8. UV-Vis spectrum of **2** (50 μM) in mammary epithelial cell growth medium (MEGM):DMSO (200:1) over the course of 72 h at 37 °C.

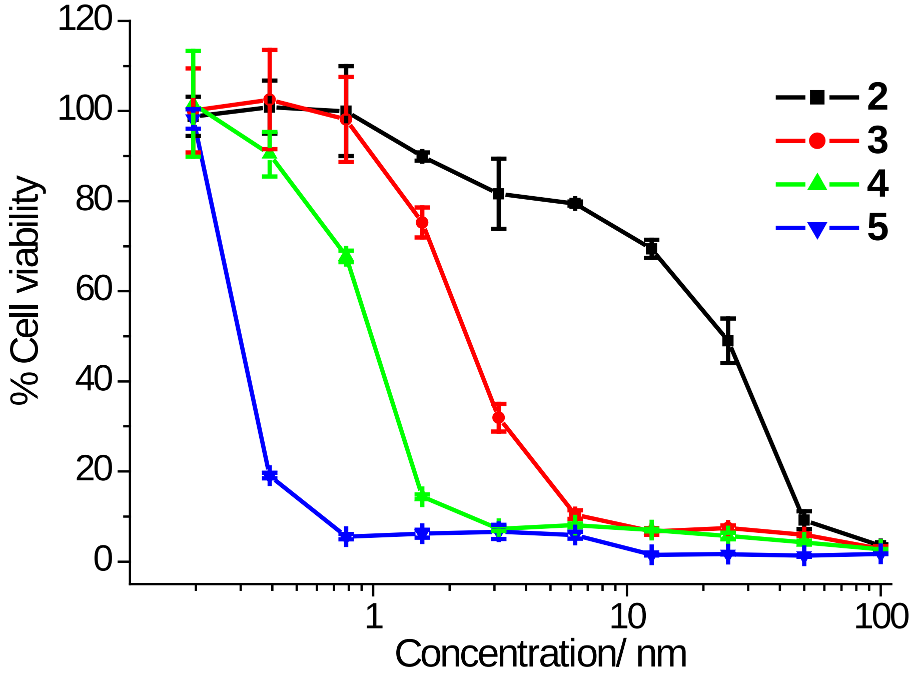

Figure S9. Representative dose response curves of **2**–**5** against HEK 293T cells after 72 h incubation.
